# Neuromuscular electrical stimulation for facial wrinkles and sagging: The 8‐week prospective, split‐face, controlled trial in Asians

**DOI:** 10.1111/jocd.16403

**Published:** 2024-07-11

**Authors:** Jun Omatsu, Takashi Yamashita, Toko Mori, Yurika Osuji, Ruriko Kawanabe, Ai Kuzumi, Asako Yoshizaki, Tomomi Yokota, Kentaro Yamazaki, Shinichi Sato, Ayumi Yoshizaki

**Affiliations:** ^1^ Department of Dermatology University of Tokyo Graduate School of Medicine Tokyo Japan; ^2^ Face Lift Laboratory, Research and Development Department YA‐MAN Ltd Tokyo Japan; ^3^ Department of Clinical Cannabinoid Research University of Tokyo Graduate School of Medicine Tokyo Japan

**Keywords:** anti‐aging, blood flow, neuromuscular electrical stimulation, sagging, wrinkles

## Abstract

**Objective:**

This study aims to fill the knowledge gap regarding the effects of high frequency facial neuromuscular electrical stimulation (fNMES) on facial aging, using a device equipped with CERTEC (Cell Energy Regeneration Technology) operating between 40 and 190 kHz.

**Methods:**

This prospective split‐face study was conducted at Tokyo University Hospital between March and May 2023 with 24 healthy adult women aged 30–59. The intervention group used the fNMES device along with basic skin care on one side of the face, and basic skin care alone on the other side for 8 weeks. Evaluations included changes in skin wrinkles, sagging, and blood flow.

**Results:**

This study found significant improvements in skin elasticity and degree of wrinkles in the areas intervened with fNMES (*p* < 0.05, respectively). In addition, the intervention resulted in significant improvements in jawline angle (*p* < 0.01), submental volume (*p* < 0.05), cheek volume (*p* < 0.05), maximum nasolabial fold depth (*p* = 0.03), and total volume of the nasolabial folds (*p* = 0.03). The fNMES intervention also showed improvement in blood flow (*p* < 0.05). These improvements were also subjectively assessed by the participants in subject questionnaires at 8 weeks after the intervention (*p* < 0.05).

**Conclusion:**

This study suggests that high frequency fNMES effectively improves facial skin elasticity, reduces wrinkles and sagging, promotes blood flow, and contributes to overall facial appearance rejuvenation. Although further studies are needed, high frequency fNMES appeared promising as a noninvasive anti‐aging therapy.

## 1 INTRODUCTION

The human face is complexly formed by the skin, subcutaneous tissue, and skeletal structure. Facial aging is caused by the transformation of these components.^1^ Known factors contributing to this process include gravity, progression of bone resorption, decreased tissue elasticity, reduction in collagen fibers, and muscle weakening on the face.^1^ These lead to changes in skin texture due to decreased elasticity, the formation of wrinkles, and sagging skin, manifesting as facial aging.^2^ Various treatments exist to counteract facial aging, ranging from surgical interventions to non‐invasive therapies.^3^ Among these, Neuromuscular Electrical Stimulation (NMES) has shown promise as a non‐invasive treatment. It induces repetitive muscle contractions through transcutaneous electrical currents.^4^

NMES is initially used in rehabilitation therapy to restore muscle strength in the abdomen and quadriceps muscles.^5^ Used on the face, it induces movement of facial muscles, which is called facial neuromuscular electrical stimulation (fNMES), and is also used in the treatment of idiopathic facial paralysis^6^, ^7^ and depression.^8^ In addition to improving these pathological conditions, fNMES is expected to have anti‐aging effects on the face by enhancing muscle thickness and counteracting age‐related muscle loss.^4^ Also, a recent study found significant improvements in skin elasticity, sagging, and double chin lifting.^9^

There are various electrical stimulation frequency bands for NMES: the extremely low frequency wave (ELF) and super low frequency wave (SLF) frequency bands below 3–300 Hz, and the kilohertz frequency band.^10^ ELF and SLF are mainly used to improve muscle strength in the trunk and extremities. On the other hand, the kilohertz frequency band has been proposed to be effective for facial aging.^9^ However, only studies using very low frequency waves at 4 kHz have been performed, and there are no studies of fNMES at higher kilohertz frequencies.^9^ YA‐MAN Ltd. (Tokyo, Japan) has found that electrical stimulation in the 10 kHz to under 200 kHz range offers a different stimulation experience compared to very low frequency waves by controlling the output frequency and distance between electrodes. (Japanese Patent Application No.2023‐220 654) This electric stimulation technology has been named CERTEC (Cell Energy Regeneration Technology) for its approach to improving sagging skin (International Application No. PCT/JP2024/000870).

This study aims to fill the knowledge gap regarding the impact of high frequency fNMES on facial aging. It evaluates wrinkles, skin sagging, and blood flow using a device equipped with CERTEC (Cell Energy Regeneration Technology) in the 40–190 kHz range. This research is expected to provide new insights into non‐invasive anti‐aging therapies.

## 2 MATERIALS AND METHODS

### 2.1 Study design and participant enrolment

This study conducted a prospective, single‐blind, split‐face study on 24 healthy adult women at Tokyo University Hospital in Tokyo, Japan, from March to May 2023. The main exclusion criteria were those with a history of hypersensitivity or contact dermatitis to the materials used in beauty devices, those who were pregnant or breastfeeding, those with malignant tumors, and those with significant differences in skin conditions or symptoms on both sides of the face. All volunteer participants were informed about the study orally and in writing, and written consent was obtained. This study was conducted by the Declaration of Helsinki and approved by the Research Ethics Committee of the Faculty of Medicine of the University of Tokyo (approval 2023001P). It is registered in UMIN‐CTR (UMIN000050696).

### 2.2 Intervention

Participants used an fNMES device and basic cosmetics on the right side of the face and only basic cosmetics on the left side for 2 months based on previous research.^11^ The fNMES device used in this study (22F1XX, YA‐MAN Ltd., Japan) outputs electrical stimulation within electromagnetic waves from 40 to 190 kHz (the aforementioned CERTEC), also featuring iontophoresis, LED light sources (wavelengths of 464–470 nm, 585–595 nm, 619–631 nm), and a cooling function. Detailed usage of this device is described in Figure S1. During the trial, participants were instructed to avoid unique skin care treatments that could affect the test area, excessive UV exposure such as sea bathing, mountaineering, sunbathing, and outdoor exercises, to take measures against UV rays, not to start taking new supplements, and to continue using the same sunscreen product (excluding topical agents and medications intended for skin quality improvement) throughout the trial period.

### 2.3 Efficacy and safety evaluation

Evaluations were conducted at 4 and 8 weeks. The evaluation parameters included the measurement of wrinkles, sagging, spots, skin hydration, and skin elasticity using measurement devices, subjective evaluations by dermatologists based on facial photographs, patient self‐assessment, and safety assessments.

### 2.4 Device‐based evaluation

The firmness/elasticity of the skin on both cheeks was measured using a Cutometer MPA580 (Courage + Khazaka Electronic GmbH, Cologne, Germany) equipped with 2 mm/6 mm probes, with a 2‐s suction under 400 mbar pressure followed by a 2‐s measurement.^12^ The skin's resistance to negative pressure (stiffness) and its ability to return to its original position (elasticity) are displayed as a real‐time measurement curve (penetration depth in mm/time). The parameters considered include R2, which represents viscoelasticity (the ratio of skin resistance during suction and the ability to return during relaxation); R5, defining net elasticity (the ratio of the elastic part during suction to the elastic portion); and R7, which is the ratio of elastic recovery to overall deformation, with results expressed in mm. The Antera 3D (version 3.1.0, Miravex, Dublin, Ireland) was used to evaluate nasolabial folds, with images analyzed using Antera 3D software.^13^ Each visit assessed the same predefined areas. Sagging was assessed using the 3D‐LifeViz Mini™ by QuantifiCare™ (Valbonne, France), recording facial data and considering decreased face line angles, submandibular volume, and cheek volume as indicators. 3D processing, landmarks, and measurements were managed with DermaPix™ software (QuantifiCare™).^14^ Tissue blood flow was evaluated using the Omegaflow FLO‐C1 laser speckle blood flow imaging system (Omegawave, Inc., Tokyo, Japan), assessing parameters such as blood flow volume, red blood cell density, and blood flow velocity.^15^

### 2.5 Dermatologist's wrinkle evaluation grade

Dermatologists evaluated wrinkles using a scale based on the wrinkle grade guidelines of the Japanese Cosmetic Science Society, with two experienced dermatologists rating crow's feet from 0 (no wrinkles) to 7 (worst) in a double‐blinded manner using photos taken with VISIA Evolution.^16^

### 2.6 Self‐evaluation questionnaires

Participants responded to each question using an 11‐point answer scale, where the state before starting was rated as 5 points at both the 4‐ and 8‐week marks (0 points: worst, 10 points: best). The questions addressed the appearance of wrinkles around the eyes, the appearance of wrinkles on the forehead, the noticeability of nasolabial folds, sagging from the cheeks to the mouth area, the feeling of a more defined jawline, and the feeling of firmness.

### 2.7 Safety assessments

The safety assessment was conducted through a dermatological assessment based on parameters of erythema, dryness, edema, urticaria, and allergic reactions. Patients were provided with forms to record any adverse events or local skin irritations observed during the study period.

### 2.8 Statistical analysis

To determine the appropriate sample size for the current study, we set the null hypothesis of no difference in skin quality between the intervention and control groups. Based on data from a Japanese prior related study, the expected standard deviation for skin elasticity was 0.07, and the anticipated difference in means between groups was 0.05–0.06. Assuming a 95% confidence interval and 80% power, the required sample size to detect a significant difference would be 18 participants per group. However, accounting for an expected 20% dropout rate, we set the total sample size at 24 participants (12 per group) to ensure adequate statistical power. Furthermore, to minimize the influence of individual skin quality differences, we chose to use a within‐subject, split‐face design, where each participant serves as their own control. This approach is expected to provide more precise and reliable results compared to a between‐subject design. The analysis was based on the Full Analysis Set, and values were presented as mean ± standard deviation (mean ± standard error for cutometer and crow's feet measurements). Comparisons were made using paired *t*‐tests (Wilcoxon signed‐rank tests for crow's feet), with significance set at a risk level of less than 5% (*p* < 0.05). Analyses were performed using SAS® version 9.4 and Prism (10.0).

## 3 RESULTS

### 3.1 Participants

The average age of participants was 45.21 ± 1.34 years (range 35–55). All 24 enrolled patients completed the study without any treatment‐related adverse events leading to discontinuation. There were no significant differences in baseline facial measurements between sides.

### 3.2 Improvement in skin elasticity from epidermis to deep dermis in the intervention group

In the Cutometer device, R2 represents viscoelasticity, R5 indicates net elasticity (excluding the influence of viscosity), and R7 denotes elasticity.^12^ A 2 mm diameter Cutometer reflects the characteristics from the epidermis to the superficial dermis, while a 6 mm diameter Cutometer reflects those from the superficial to the deep dermis.^12^ Changes from baseline at 4 and 8 weeks were demonstrated for R2, R5, and R7 (Figure 1). From the 2 mm results, significant improvements were noted at 4 weeks in the intervention group compared to the control group for R2 (5.08 ± 3.79% vs. −5.92 ± 4.03%, *p* < 0.01), R5 (7.06 ± 3.54% vs. −6.00 ± 3.60%, *p* < 0.01), and R7 (11.61 ± 4.60% vs. −2.48 ± 4.94%, *p* < 0.01), with all parameters showing improvement from baseline. Similar improvements were observed at 8 weeks for R2 (10.18 ± 5.03% vs. −8.14 ± 4.59%, *p* < 0.01), R5 (2.31 ± 3.48% vs. −15.84 ± 2.83%, *p* < 0.01), and R7 (10.61 ± 5.70% vs. −9.73 ± 4.99%, *p* < 0.01). From the 6 mm results, significant improvements were also noted at 4 weeks in the intervention group for R2 (−0.52 ± 2.00% vs. −10.54 ± 2.08%, *p* < 0.01), R5 (−14.77 ± 2.10% vs. −23.50 ± 2.09%, *p* < 0.01), and R7 (−5.38 ± 1.64% vs. −16.31 ± 1.78%, *p* < 0.01), with a continuing trend of improvement at 8 weeks for R2 (2.34 ± 2.24% vs. −11.52 ± 1.87%, *p* < 0.01), R5 (−12.72 ± 2.60% vs. −26.01 ± 2.40%, *p* < 0.01), and R7 (−3.76 ± 2.58% vs. −19.40 ± 1.97%, *p* < 0.01). While spots and skin hydration were also measured, no significant differences were observed between the intervention and control groups for these parameters.

**FIGURE 1:**
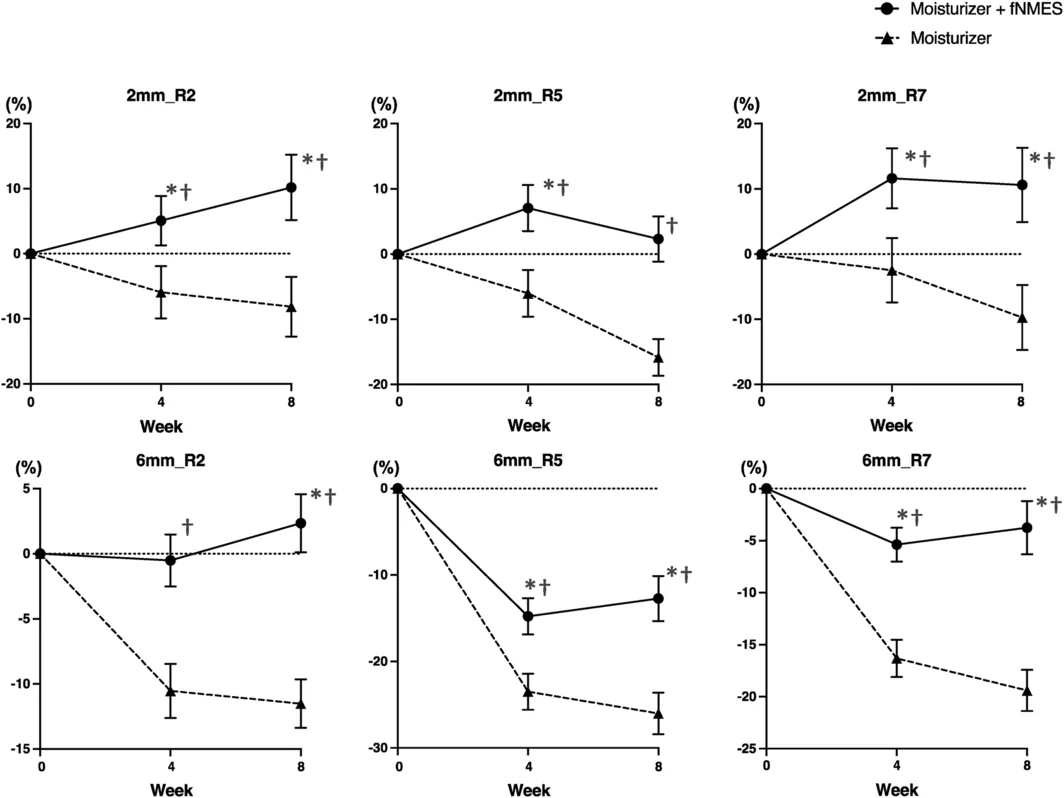
Changes in R2, R5, and R7 from baseline (%) at 4 and 8 weeks using 2 mm or 6 mm diameter Cutometer devices, *N* = 24. Error bars represent standard error of the mean. *p < 0.05 compared to baseline. †*p* < 0.05 when compared between moisturizer + fNMES and moisturizer.

### 3.3 Improvement in objective wrinkle grade assessment in the intervention group

The wrinkle grade assessment is an evaluation method used for the functional assessment of products targeting crow's feet wrinkles in Japan.^16^ Data from dermatologist‐conducted wrinkle grade assessments were obtained for 24 participants (Figure 2A,B). There were no significant differences between the two groups at baseline and 4 weeks. However, a significant difference was noted between the groups at 8 weeks (intervention group 2.67 ± 0.24, control group 3.50 ± 0.35, *p* = 0.002). Furthermore, the intervention group demonstrated significant improvements compared to the baseline (0 weeks: 3.67 ± 0.32; 8 weeks 8 2.67 ± 0.24, *p* = 0.001), indicating the efficacy of the device in improving wrinkle grades.

**FIGURE 2:**
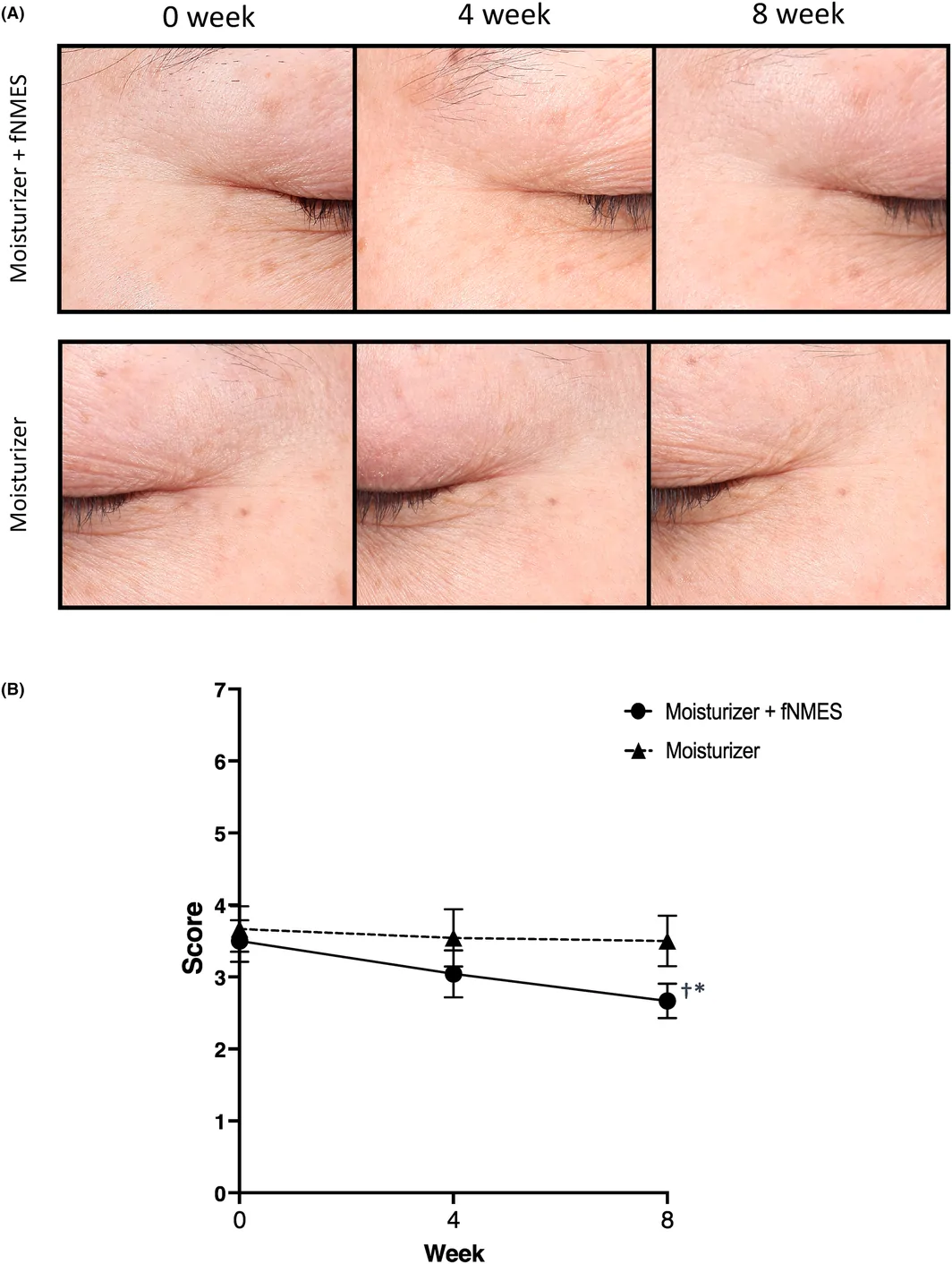
(A) Representative close‐up photographs of the crow's feet area at baseline, 4 and 8 weeks showing changes on the right side of the face (moisturizer + fNMES) and the left side of the face (moisturizer only). (B) Wrinkle assessment grade scores for the crow's feet area from baseline to 8 weeks for the left and right sides. Error bars represent the standard error of the mean. *p < 0.05 compared to baseline. †*p* < 0.05 for the comparison between moisturizer + fNMES and moisturizer only.

### 3.4 Improvement in jawline angle, submental volume, and cheek volume in the intervention group

Changes in jawline angle, submental volume, and cheek volume were assessed using 3D images captured with the 3D‐LifeViz Mini™. Representative photographs at baseline and 8 weeks with jawline angle are shown (Figure 3A) (Representative raw photographs are shown in the Figure S2). In the intervention group, significant improvements were observed in the jawline angle at both 4 and 8 weeks compared to the control group (4 weeks: intervention group −2.41 ± 0.42°, control group 0.01 ± 0.37°, *p* < 0.01; 8 weeks: intervention group −2.32 ± 0.35°, control group 0.35 ± 0.36°, *p* < 0.01; Figure 3B). The amount of change in facial volume from baseline at 8 weeks was identified on a color scale (Figure 3C). Analysis of submental volume and cheek volume showed no difference from baseline at 4 weeks but a significant difference at 8 weeks in the intervention group (submental volume change: intervention group −0.93 ± 0.30 cm^3^, control group −0.32 ± 0.26 cm^3^, *p* < 0.05; cheek volume change: intervention group −1.15 ± 0.37 cm^3^, control group −0.50 ± 0.23 cm^3^, *p* < 0.05; Figure 3D,E).

**FIGURE 3:**
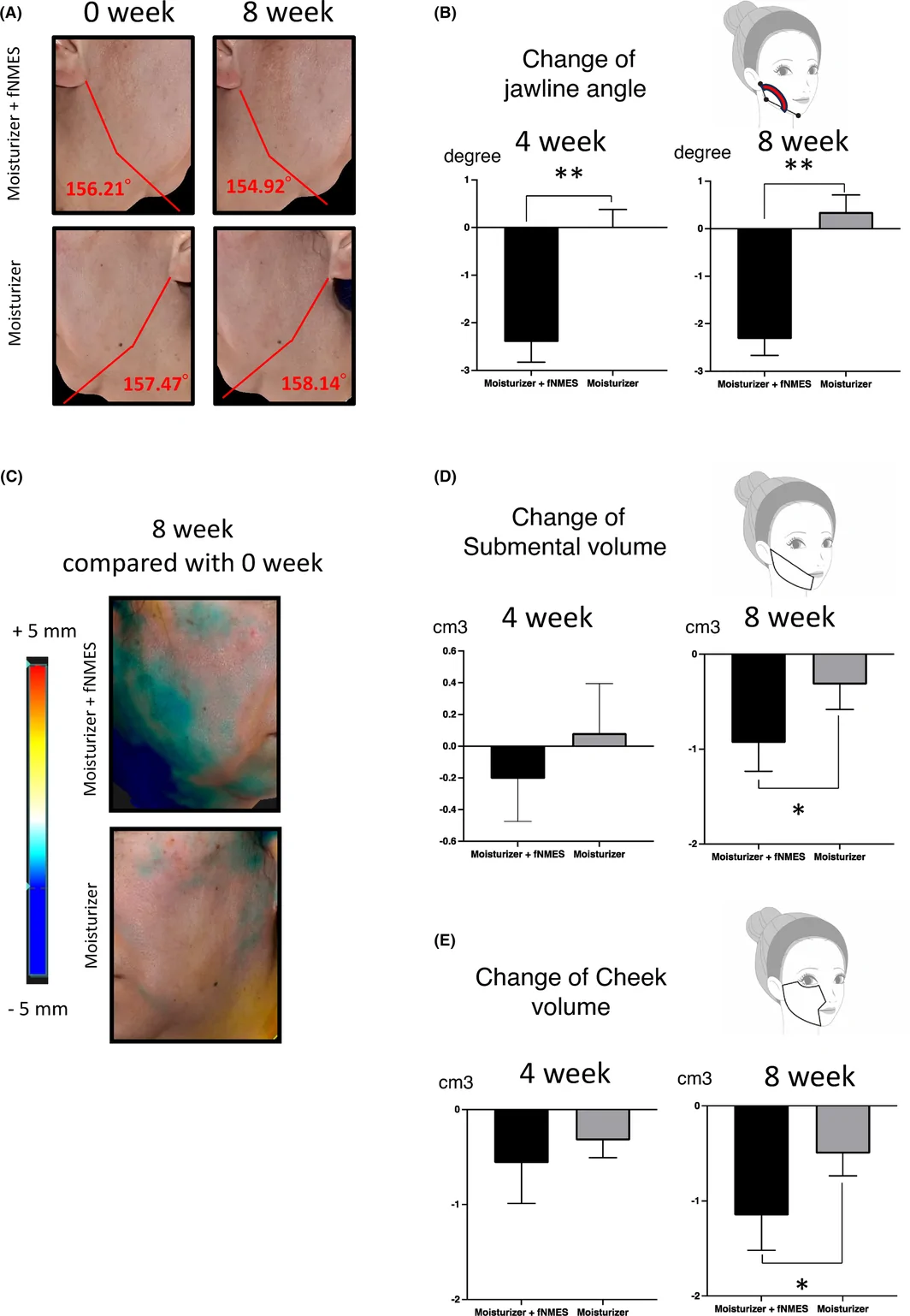
Measurement results at 4 and 8 weeks for intervention group and control group using 3D‐LifeViz Mini™. (A) Representative photo at baseline and 8 weeks with jawline angle automatically calculated by the instrument (right cheek: moisturizer + fNMES, left cheek: (moisturizer only). (B) The difference in the angular change of the jawline from the baseline (left: after 4 weeks, right: after 8 weeks). (C) Representative photo at 8 weeks (right cheek: moisturizer + fNMES, left cheek: moisturizer only). Volume changes from the baseline are indicated in color scale. (D) The difference in submental volume from the baseline (left: after 4 weeks, right: after 8 weeks). (E) The difference in cheek volume from the baseline (left: after 4 weeks, right: after 8 weeks). Error bars represent standard error of the mean. *p < 0.05, **p < 0.01, indicating statistical significance compared between moisturizer + fNMES and moisturizer.

### 3.5 Improvement in nasolabial folds in the intervention group

The Antera 3D facial imaging device measured changes in the nasolabial folds. Representative photographs of the nasolabial folds at baseline and 8 weeks are presented in Figure 4A, showing changes for both the intervention (moisturizer + fNMES) group and the control (moisturizer only) group (Representative raw photographs are shown in the Figure S3). There were no significant differences in total volume and maximum depth of nasolabial folds at baseline (Figure 4B). The intervention group exhibited a significant reduction in the total volume change compared to the control group at 8 weeks (intervention group −0.25 ± 0.72 cm^3^, control group 1.15 ± 0.54 cm^3^, *p* = 0.03; Figure 4C). The change in maximum depth also showed a significant decrease at 8 weeks (intervention group −0.027 ± 0.016 mm, control group 0.008 ± 0.014 mm, *p* = 0.03; Figure 4D).

**FIGURE 4:**
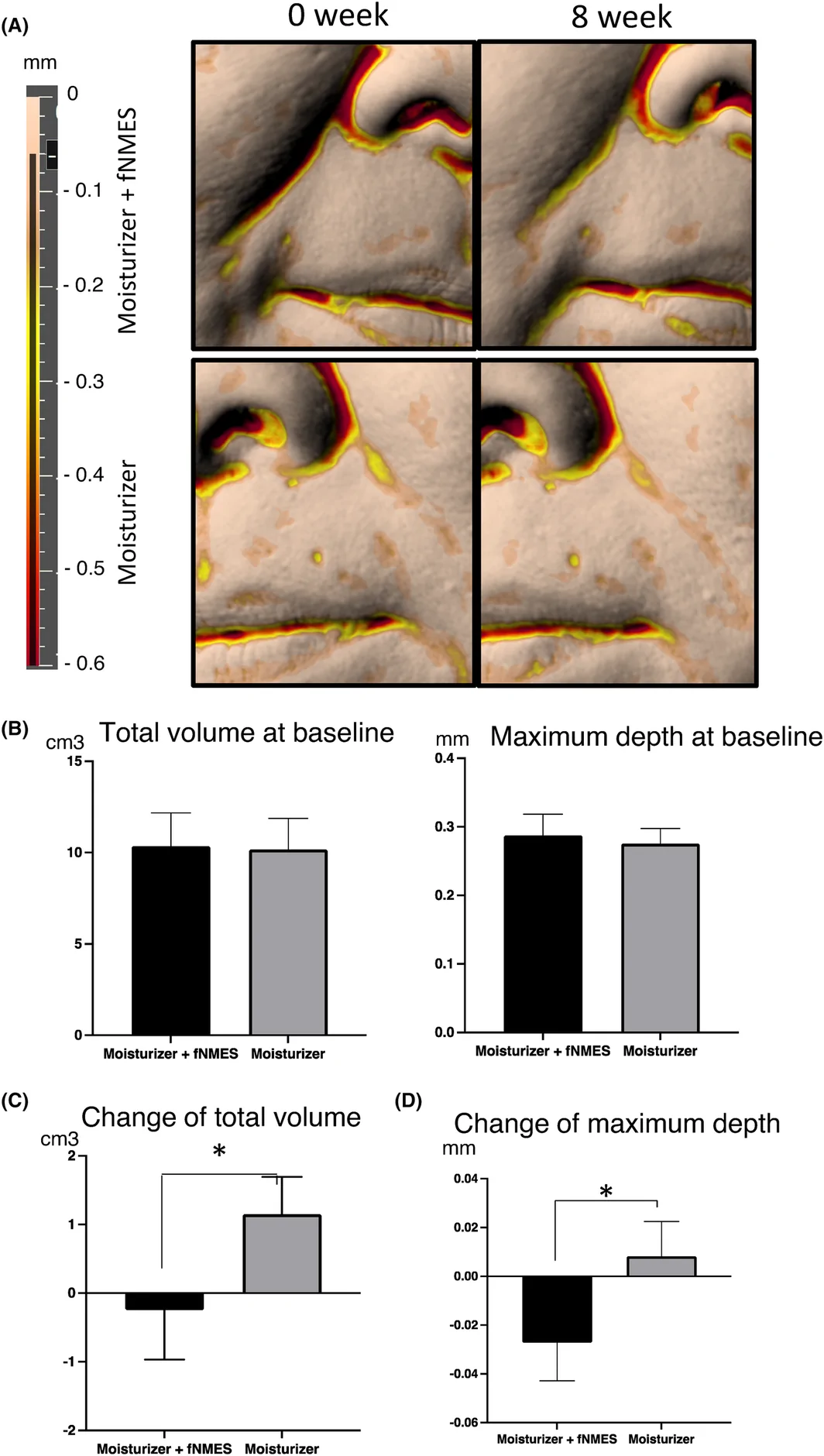
Measurement of the total volume and maximum depth of nasolabial folds using the Antera 3D facial imaging device. (A) Representative photo at baseline and 8 weeks. Depth is indicated by a red grayscale. (right cheek: moisturizer + fNMES, left cheek: (moisturizer only). (B) Total volume of nasolabial folds at baseline and maximum depth at baseline are shown. (C) Changes in the total volume of nasolabial folds from baseline to 8 weeks. (D) Changes in maximum depth of nasolabial folds from baseline to 8 weeks. Error bars represent standard error of the mean. *p < 0.05 compared between moisturizer + fNMES and moisturizer.

### 3.6 Improvement in skin blood flow in the intervention group

The blood flow, blood flow velocity, and red blood cell density in the face were measured by Omegaflow FLO‐C1 laser (Figure 5). Red blood cell density showed no significant difference at the start and 4 weeks, nor was there a significant difference between the intervention and control groups. While no significant difference was observed between the intervention and control groups in tissue blood flow and blood flow velocity at 4 weeks, both tissue blood flow (intervention group 1.07 ± 0.08, control group 0.87 ± 0.05) and blood flow velocity (intervention group 1.03 ± 0.06, control group 0.91 ± 0.04) significantly increased at 8 weeks.

**FIGURE 5:**
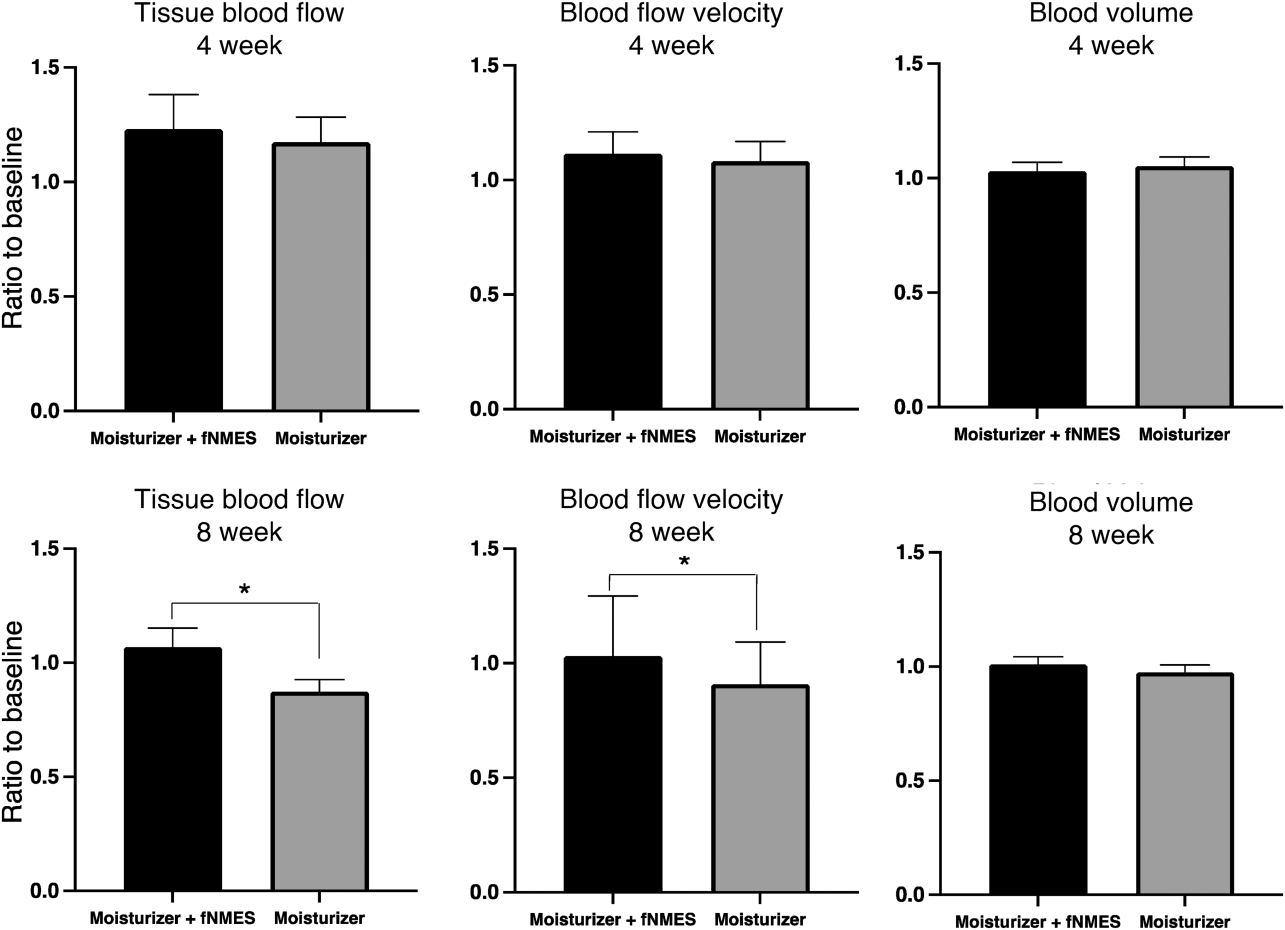
Measurement of facial skin blood flow, blood flow velocity, and blood volume (red blood cell density) using a laser speckle blood flow imaging system at 4 and 8 weeks. The bars in each graph indicate the ratio from the baseline. Error bars represent standard error of the mean. *p < 0.05 compared between moisturizer + fNMES and moisturizer.

### 3.7 Improvement in various skin indices reported in participant questionnaires in the intervention group

In the participant questionnaires, a significant increase in the feeling of firmness was observed from 4 weeks compared to the baseline (Figure 6). Similarly, improvements were noted in the appearance of wrinkles around the eyes, appearance of wrinkles on the forehead, noticeability of nasolabial folds, sagging from the cheeks to the mouth area, and the feeling of a more defined jawline. Additionally, significant improvements continued at 8 weeks for each of these items (Figure 6). This demonstrated that in the device intervention group, improvements were observed not only in objective measures but also in subjective indicators.

**FIGURE 6:**
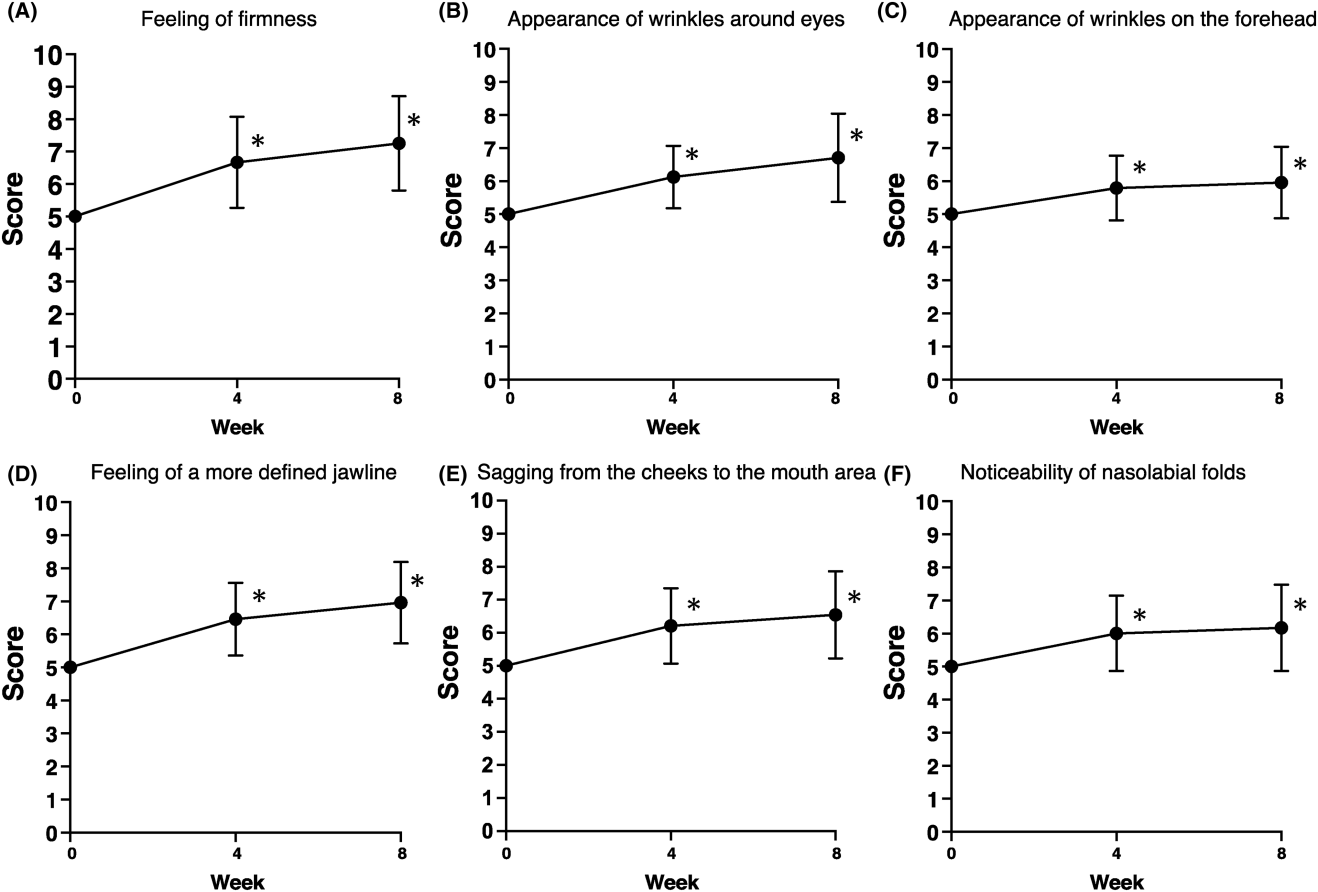
The results of participant questionnaires at 4 and 8 weeks, using 5 points as the reference point, are shown. The items are (A) feeling of firmness, (B) appearance of wrinkles around the eyes, (C) appearance of wrinkles on the forehead, (D) sagging from the cheeks to the mouth area, (E) feeling of a more defined jawline, and (F) noticeability of nasolabial folds. Error bars represent the standard deviations. *p < 0.05 compared to baseline.

### 3.8 Adverse effects

No adverse events were observed in either group.

## 4 DISCUSSION

This study is the first to demonstrate that facial treatment using a high frequency fNMES device can improve wrinkles, sagging, and blood flow in facial skin. A comparison between the side of the face treated with the high frequency fNMES device and the untreated side showed improvements at 8 weeks in objective measures such as elasticity (Figure 1), wrinkle grade (Figure 2), jawline angle, submental volume, cheek volume (Figure 3), and total volume and the maximum depth of nasolabial folds (Figure 4). In addition, the decrease in blood flow was reduced on the treated side compared to the control side (Figure 5). Furthermore, subjective participant evaluations also showed improvement at both 4 and 8 weeks (Figure 6).

Facial aging is caused by a complex interplay of factors, including gravity, progressive bone resorption, decreased tissue elasticity, reduction in collagen fibers, and muscle weakness, leading to changes in skin texture, wrinkle formation, and sagging, which are manifestations of facial aging.^17^ Various factors are involved in the formation of wrinkles. In particular, loss of skin elasticity is considered a significant factor. As age increases, collagen and elastin in the skin decrease and undergo structural changes, resulting in reduced skin elasticity, manifesting as wrinkles.^1^ This study revealed that the group using the high frequency fNMES device improved skin viscoelasticity, net elasticity, and elasticity, as measured by the Cutometer, compared to the control group (Figure 1). These three characteristics reflect the skin's ability to return to its original state and are known to decrease with age.^12^ In this study, these three properties in the epidermis to superficial dermis decreased in the control group compared to the baseline. In contrast, in the intervention group, they improved compared to both the baseline and the control group. Moreover, these properties also significantly improved in the superficial to deep dermis compared to the control group. These results suggest that the high frequency fNMES device effectively improves elasticity across various layers of the skin, from the epidermis to the deep dermis. In the wrinkle grade evaluation conducted by doctors, which directly and objectively assesses crow's feet, the high frequency fNMES device group showed a significant improvement in scores compared to the control group (Figure 2). The mechanism by which fNMES improves skin elasticity has not yet to be clarified. However, previous studies indicate that an 8‐week facial muscle exercise program for women in their 30s–50s significantly increased skin viscoelasticity, net elasticity, and elasticity.^18^ Considering that the high frequency fNMES device induces involuntary facial muscle contractions, passive facial muscle movement could improve skin elasticity and reduce wrinkles through a similar mechanism.

Sagging also appears due to a combination of factors. The formation of sagging involves the reduction and structural changes in collagen and elastin leading to decreased skin elasticity, weakening of facial muscles, and increased subcutaneous fat.^1^ As shown in Figure 1, skin elasticity improved in the group using the high frequency fNMES device. Moreover, significant improvements in jawline angle, submental volume, and cheek volume were observed compared to the control group, as revealed by the examination of 3D images obtained with the 3D‐LifeViz Mini™ (Figure 3). This may indicate a reduction in subcutaneous fat along the jawline. In addition, both the total volume and maximum depth of nasolabial folds significantly decreased in the high frequency fNMES device group compared to the control group (Figure 4). It suggests the device's comprehensive effect in improving elements of sagging. As mentioned earlier, the weakening of facial muscles is one factor in sagging. A positive correlation is reported between the reduction in the temporalis and masseter muscle activity during chewing and maximum occlusion and sagging.^19^ Previous studies have shown an increase in the measured thickness of the zygomatic major, one of the facial expression muscles, after 12 weeks of low frequency fNMES,^20^ suggesting that high frequency fNMES might also improve sagging by increasing facial muscle mass. An increase in subcutaneous fat is also caused of sagging. An increase in subcutaneous fat not only makes the skin more susceptible to gravitational effects, but enlarged fat cells also increase the expression of MMP9 through ERK signaling, breaking down elastic fibers in the dermis and reducing its elasticity.^21^ Adiponectin, a cytokine specifically secreted by fat cells, increases the secretion of hyaluronic acid and collagen from dermal fibroblasts.^22^ On the other hand, adiponectin secretion is decreased in obese individuals.^23^ Animal study shows obesity reduces dermal fibroblasts and decreases dermal thickness and elasticity.^24^ Therefore, reducing subcutaneous fat becomes important in improving sagging. Moreover, exercising muscles also reduces surrounding adipose tissue.^25^ The high frequency fNMES device in this study likely reduced surrounding adipose tissue by passively moving facial muscles. The device has an effect in improving sagging through such a complex approach.

Aging is associated with blood flow in the skin.^26^, ^27^ A decrease in skin blood flow can lead to the accumulation of oxidants, causing tissue damage.^28^ Numerous reports on NMES and skin blood flow have established its effect in increasing blood flow.^29^, ^30^ A study comparing low and high frequency NMES has shown that high frequency NMES significantly increases skin temperature compared to low frequency NMES.^31^ In this study, the high frequency fNMES device group showed significantly improved blood flow volume and velocity compared to the control group (Figure 5). Increased skin blood flow has an anti‐aging effect on skin aging and high frequency fNMES could be expected to have this effect.

In this study, subjective evaluations of facial skin condition by participants also greatly improved (Figure 6). Firmness succinctly expresses the state of skin elasticity, and its improvement reflects the enhancement of skin elasticity. Additionally, the improvements in crow's feet wrinkles (Figure 2), nasolabial fold wrinkles (Figure 3), and sagging of the jawline (Figure 4) observed in objective measurements were also confirmed in subjective evaluations.

The assessments were taken at two time points in this study, which were 4 and 8 weeks after the intervention. At 4 weeks, an improvement in skin viscoelasticity was observed, but no significant differences were noted in other items (Figures 2, 3 and 5). At 8 weeks, however, many items showed significant differences compared to the control group. This suggests that the effect of the high frequency fNMES device is not permanent but temporary. Therefore, continuous intervention is necessary to achieve the improvement effect of wrinkles and sagging.

The fNMES device used in this study has additional features beyond electrical muscle stimulation, such as iontophoresis and LED light sources (wavelengths of 464–470 nm, 585–595 nm, and 619–631 nm). Iontophoresis has been shown to enhance transdermal delivery of active ingredients, potentially improving the penetration and efficacy of the conductive lotion used in conjunction with the device.^32^ Similarly, the LED light sources at specific wavelengths have been associated with various skin benefits. While the primary intervention in this trial was fNMES stimulation, these complementary modalities may have contributed to the observed improvements in wrinkles and sagging. In particular, the LED light at wavelengths of 585–595 nm and 619–631 nm have been associated with increased collagen production, improved circulation, and reduced inflammation.^33^, ^34^ Additionally, blue light at 464–470 nm has been suggested to affect nitric oxide metabolism, leading to better perfusion of tissues and promoting mitochondrial recovery.^35^ These effects could have potentially complemented the primary fNMES intervention in reducing wrinkles and sagging. However, it should be noted that the LED light sources in the device used in this study had lower output compared to those used in other studies, which may have limited the additional benefits of the LEDs. Many previous studies have utilized LEDs with higher output and longer exposure times.^33^, ^36^, ^37^ Therefore, it is possible that the observed improvements in wrinkles and sagging were influenced by the synergistic effects of iontophoresis and LED light exposure acting as adjunctive therapies. Further investigation may be warranted to elucidate the specific contributions of each component and optimize the treatment protocol for optimal facial rejuvenation results.

This study revealed that high frequency fNMES can improve wrinkles, sagging, and blood flow in the skin and can be used safely. However, there are several limitations to this study. First, there might be a selection bias due to the small number of participants. All registered patients had the same ethnic background. Limiting the device use area to the right cheek without randomization might introduce bias due to anatomical differences between the cheeks. This study involved the use of fNMES for 8 weeks, and future long‐term follow‐up studies are needed to investigate the long‐term efficacy and duration of fNMES effects. Despite these limitations, this study demonstrates the effectiveness of high frequency fNMES in improving wrinkles, sagging, and blood flow in facial skin, using not only subjective evaluations by participants but also objective indicators such as 3D images. These findings suggest that fNMES may represent an effective and safe adjunctive option for facial rejuvenation treatments. While potential risks associated with NMES, such as heating, drying, and pigmentation changes, are generally considered, no such adverse events were observed in the current investigation. Although further evaluation may be warranted, the usage protocol employed in this study suggests that fNMES could potentially be utilized as a safe modality.

## AUTHOR CONTRIBUTIONS

This work was a collaborative effort. All authors discussed the results and contributed to the final manuscript.

## CONFLICT OF INTEREST STATEMENT

The authors declare that they have no conflicts of interest to disclose.

## ETHICS STATEMENT

This study was conducted in accordance with the ethical standards of the institutional and national research committee and with the 1964 Helsinki declaration and its later amendments or comparable ethical standards. Informed consent was obtained from all individual participants included in the study. All procedures performed in studies involving human participants were in accordance with the ethical standards of the University of Tokyo and with the 1964 Helsinki declaration and its later amendments or comparable ethical standards.

## Supporting information


**Figure S1.** The schematic diagram shows how to apply the device. After performing their regular basic skincare routine, the subjects applied a conductive lotion, which they typically used, to both sides of the face. The fNMES device was then used on the right side of the face. No additional conductive gels or media were employed beyond the subjects’ regular lotion. The subjects were instructed to use the fNMES device on the right side of the face following a typical usage regimen over a 7‐day cycle. Specifically, for 3 days of the cycle, they used the device for 7 min once daily. On 2 other days, they used it for 2.5 min once daily, and on the remaining 2 days, they used it for 1.5 min once daily. This usage protocol was designed to mimic real‐world conditions and practices for at‐home fNMES facial treatment devices. Subjects are applied in the order and direction according to the schematic diagram from 1 to 5. The shaded areas on the schematic diagram, specifically the upper and lower eyelids, were not treated with the device in this study.
**Figure S2.** This is a supplemental figure to Figure 3, which was measured using the 3D‐LifeViz Mini™. Representative raw images taken from the front, right side (moisturizer + fNMES) and left side (moisturizer only) at baseline and 8 weeks are shown.
**Figure S3.** This is a supplemental figure to Figure 4, which was measured using the Antera 3D facial imaging device. Representative raw images taken from right side (moisturizer + fNMES) and left side (moisturizer only) at baseline and 8 weeks are shown.

## Data Availability

The data that support the findings of this study are available on request from the corresponding author. The data are not publicly available due to privacy or ethical restrictions.
