# Correction to “Neuromuscular Electrical Stimulation for Facial Wrinkles and Sagging: The 8‐Week Prospective, Split‐Face, Controlled Trial in Asians”

**DOI:** 10.1111/jocd.70786

**Published:** 2026-03-15

Omatsu J, Yamashita T, Mori T, et al. Journal of Cosmetic Dermatology. 2024; 23:3222–3233. DOI: 10.1111/jocd.16403

In the originally published version of this article, the Conflict of Interest disclosure was incomplete, and the ethics approval number was stated incorrectly.

Specifically, employment relationships with YA‐MAN Ltd. and the provision of study devices and measurement instruments were not fully disclosed in the Conflict of Interest statement. In addition, the ethics approval number was incorrectly reported as *2023001P*.

Correction

#Conflict of Interest

Tomomi Yokota and Kentaro Yamazaki are employees of YA‐MAN Ltd.

YA‐MAN Ltd. provided some of the facial devices and measurement instruments used in this study.

The other authors declare no conflicts of interest.

#Ethics approval

The correct ethics approval number is *2022299NI*.

These corrections do not affect the results or conclusions of the study.

We apologize for this error.

